# Overdosage Section in US and EU Labeling

**DOI:** 10.1007/s43441-024-00673-y

**Published:** 2024-06-17

**Authors:** Sarah Condon, Thomas G. Cantu, Antony Constantinou, Erin C. Degnan, Monica Lungu, Marcella G. Paglione, Shreya J. Parikh, Joanna Szewczyk

**Affiliations:** 1 GSK, London, UK; 2 GSK, Collegeville, PA USA; 3 GSK, Wavre, Belgium

**Keywords:** Overdosage, Labeling, Safety, Regulatory Affairs

## Abstract

The Prescribing Information (PI) in the United States (US) and the Summary of Product Characteristics (SmPC) in the European Union (EU) are approved by the US Food & Drug Administration (FDA), and the European Medicines Agency (EMA), respectively. The inclusion of overdosage information in these documents is a regulatory requirement in both regions. This research evaluates the content of the overdosage section of US and EU labeling. The overdosage sections of labels for drugs approved in the US in three time periods were analyzed: 2000–2001, 2010–2011, and 2020–2021. EU labels for these same products were also reviewed if registered through the Centralized Procedure. Data collection and analyses were performed using a predefined questionnaire, focusing on adherence to regulatory requirements and identifying areas where additional regulatory guidance may be beneficial. The findings indicate that the content of the overdosage sections largely comply with the regulatory requirements of their respective regions. Fewer than half of the labels included information on supratherapeutic doses observed from clinical studies, risk factors for overdose or population specific data associated with overdose. Inconsistencies were noted concerning the incorporation of animal data when human data were available, in addition to the referencing of Poison Centers. The overall utility of non-specific treatment recommendations, in addition to gastric lavage is discussed. While the content of the overdosage section generally aligns with regulatory expectations, additional regulatory guidance could enhance consistency in how this section of labeling is presented and clarify expectations to improve its usefulness for health care professionals (HCPs).

## Introduction

The PI in the US and the SmPC in the EU are approved as part of the marketing authorization of each medicine by the US FDA and the EMA, respectively. These documents form the basis of information for HCPs and include the benefits and risks of the drug when used for approved indications, pharmaceutical information, and information for individualized patient care (e.g., pediatric populations, and patients with renal or hepatic impairment). Marketing authorization holders (MAHs) are responsible for ensuring that the label is accurate and reflects the most current knowledge about the safety and efficacy of the drug. Reference to unapproved dosing regimens should generally be avoided in labeling, as this may promote “off-label” dosing. Unapproved dosing regimens may be included in labeling if it is in the context of noting a lack of incremental benefit or increased toxicity with higher doses. The overdosage section of the US PI (Sect. 10) and the overdose section of the EU SmPC (Sect. 4.9) is intended to inform prescribers of anticipated signs and symptoms of drug overdose and how to monitor and treat patients who may experience an overdose.

US regulations, specifically US 21 CFR 201.57(c)(11) and US 21 CFR 201.80(i), set forth the requirements governing the content and format of labeling within the overdosage section of the US PI, albeit with differing applicability [1, 2]. The “Physician’s Labeling Rule” (US 21 CFR 201.57), including subsection (c)(11) relating to overdose, applies to drugs which were approved on, or after June 30, 2001 [1]. It also applies to drugs approved before June 30, 2001 if the MAH submits an efficacy supplement after June 30, 2006. Additionally, MAHs have the option to voluntarily update their labeling to comply with 21 CFR 201.57. US 21 CFR 201.80(i) is applicable to drugs approved prior to June 30, 2001 [2]. Unlike many other sections within US labeling, there exists no official guidance issued by the FDA concerning the content and format of the overdosage section. As per US 21 CFR 201.56(d)(4) and US 21 CFR 201.56(e)(3), there is the option to omit the overdosage section of the US PI where there is an absence of information [1, 2]. In the EU, Article 11 of Directive 2001/83/EC establishes the legally binding requirements governing the content and format for the overdose section within the EU SmPC [3]. In contrast to the US, various guidance documents are available in the EU to aid in adhering to these requirements:

1. Notice to Applicants (NtA): A Guideline on SmPC: Offers guidance on the presentation of overdose information in the label [4].
2. Quality Review of Documents (QRD) template: Offers practical guidance on creating the SmPC, including recommended headers [5].
3. SmPC Advisory Group training presentations: Incorporates illustrative examples, pertaining to Sect. 4.9 and a FAQ [6].

The inclusion of Sect. 4.9 of the EU SmPC is mandatory as stated in Article 11 of Directive 2001/83/EC [3]. Summaries of the US FDA and EMA regulations and/or guidelines for the overdosage section of labeling are provided in Tables 1 and 2.

**Table 1:** Labeling requirements for the overdosage section of the US PI

US 21 CFR 201.57(c)(11) [1]	US 21 CFR 201.80(i) [2]
*10 Overdosage* This section must be based on human data. If human data are unavailable, appropriate animal and in vitro data may be used. The following specific information must be provided: (i) Signs, symptoms, and laboratory findings associated with an overdosage of the drug; (ii) Complications that can occur with the drug (for example, organ toxicity or delayed acidosis); (iii) Concentrations of the drug in biologic fluids associated with toxicity or death; physiologic variables influencing excretion of the drug, such as urine pH; and factors that influence the dose response relationship of the drug, such as tolerance. The pharmacokinetic data given in the “Clinical Pharmacology” section also may be referenced here, if applicable to overdoses; (iv) The amount of the drug in a single dose that is ordinarily associated with symptoms of overdosage and the amount of the drug in a single dose that is likely to be life threatening; (v) Whether the drug is dialyzable; and (vi) Recommended general treatment procedures and specific measures for support of vital functions (e.g., proven antidotes, gastric lavage, forced diuresis, or as per Poison Control Center). Such recommendations must be based on data available for the specific drug or experience with pharmacologically related drugs. Unqualified recommendations for which data are lacking for the specific drug or class of drugs must not be stated.	The requirements included in this regulation are essentially the same as that provided in US 21 CFR 201.57 (c)(11), with the following exceptions: (i) Specific information should be provided on oral LD50 of the drug in animals, (ii) Does not mention the option of referring to Poison Centers

**Table 2:** Labeling requirements & guidances for the overdose section of the EU SmPC

Directive 2001/83/EC (3)	Notice to applicants: A guideline on SmPC (4)	QRD product information template (5)	SmPC training presentations (6)
*Article 11* The summary of the product characteristics shall contain, in the order indicated below, the following information: (..) 4.9. Overdose (symptoms, emergency procedures, antidotes).	4.9 Overdose Describe acute symptoms and signs and potential sequelae of different dose levels of the medicinal product based on all available information including accidental intake, mistakes and suicide attempts by patients. Taking into account all relevant evidence, describe management of overdose in man, e.g. in relation to monitoring or use of specific agonists/antagonists, antidotes or methods to increase elimination of the medicinal product such as dialysis. However, there should not be any dosage recommendation of other medicinal products (e.g. antidotes) as it could create conflict with the SmPCs of those other products. If applicable, counteractive measures based on genetic factors should be described. **Additional information on special populations** Information specifically observed in special populations such as elderly, patients with renal impairment, patients with hepatic impairment, other concomitant diseases etc. **Paediatric population** If there are specific paediatric considerations, there should be a sub-section entitled ‘paediatric population’. Special mention should be made of those medicinal products/strength of formulation for which ingestion of only one dose unit by children can cause fatal poisoning.	< text>: Text to be selected or deleted as appropriate.] 4.9 Overdose [Additional sub-headings, such as “Symptoms” or “Management” can be stated, if necessary.] < Paediatric population>	A training presentation for Sect. 4.9 of the label have been created by the SmPC Advisory group, which includes a number of exemplary labels pertinent to this section, in addition to a FAQ.

The primary objective of this research was to analyze the content of the overdosage section of drug labeling for products approved in the US and the EU, with a focus on adherence of drug labeling with some aspects of the regulatory requirements and identifying areas where additional regulatory guidance on the content of the overdosage section could be of value.

## Methods

### Scope of Products and Identification of Product Labeling

The products in scope of this research were all drugs approved by the Center for Drug Evaluation and Research (CDER) of the US FDA in three distinct time periods: 2000–2001, 2010–2011, and 2020–2021. The selection of approval years was guided by the desire to encompass a range of medicinal products that represent both newly approved drugs (< 5 years) and those that have been available in the market for an extended duration (10–20 years), enabling assessment of current trends in comparison to prior years. Furthermore, the research included a subset of US drugs that had also received EU authorization from the EMA through the EU Centralized Procedure.

To compile the list of FDA approved drugs within the specified timeframe, we accessed the ‘New Drugs at FDA: CDER’s New Molecular Entities and New Therapeutic Biological Products database’, which provides information regarding medicine approvals by year [7]. For products approved by CDER in the previously mentioned years, the individual drug labels were identified by referencing DailyMed [8]. This source allows for the retrieval of the current product labeling. In the case of drug products authorized by the EMA through the EU Centralized Procedure, individual product labels were identified by conducting a search for the European Public Assessment Reports (EPARs) for the active ingredient via the EMA public website [9]. EPARs provide detailed summaries of the assessment, including labeling, for medicinal products in the EU. If no EPAR was available, then the EU product was excluded from the analysis.

### Data Collection and Analyses

Upon retrieval of the current label for the identified drugs, a review of the overdosage section of each label was conducted based on a set of a pre-defined questions (Table 3), which allowed for a structured and objective analysis of the content. The questionnaire was structured to include one general information question pertaining to whether an overdosage section was present in the label, followed by an additional twelve questions that were directly relevant to the content and details contained within the overdosage section. The selection of the specific questions was determined by a review of the US and EU labeling regulations and guidances (Tables 1 and 2), in addition to the team’s consideration of comments that had arisen during the preparation of the overdosage section of labels for submission to the FDA and/or EMA. The specific questions were split into two groups ‘Overdosage Signs and Symptoms’ and ‘Overdosage Treatment’.

**Table 3:** Questionnaire employed for label content analysis

General question	Specific questions	
	Overdose signs and symptoms	Overdose treatment
Is the Overdose section present?	Are experiences from clinical studies using higher than the approved doses described? - If yes, are any Adverse Drug Reactions (ADRs) listed?	Are non-specific treatment recommendations provided?
	Does the label state that overdose ADRs are expected to be consistent with the general safety profile / pharmacologic effects of the drug?	Are specific treatment recommendations provided? If yes, what are they?
	Are specific ADRs and/or lab abnormalities from overdose provided? - If yes, are these from single or multiple doses or both or not stated? - If yes, are these unique to overdose (not listed in other sections of the label? ) - If yes, are the ADRs listed in the overdose section also listed in other sections of the label?	Is drug dialyzability addressed in overdose section? If no, is the drug dialyzable?
	Are risk factors for overdoses provided?	Is GI decontamination recommended (gastric lavage, induce vomiting, activated charcoal)?
	Are population-specific (e.g., pediatrics or elderly) experiences or treatment provided?	Is reference to contacting a Poison Center included?
	Are in vitro data provided?	
	Are animal data provided? If so, are LD50 data provided?	

In the data collection phase, eight individuals collaboratively participated in the examination and documentation of the data, employing a shared Microsoft Excel document as their working platform. The questions were designed so that they could be answered easily by navigating only to the relevant section of the label. However, to determine whether a drug was dialyzable, a set of specific criteria was applied. A drug was categorized as dialyzable if the label unequivocally stated it is dialyzable or if it met all of the following conditions [10]:

- The molecular weight of the drug was less than 500 Daltons.
- Protein binding was less than 80%.
- The volume of distribution was less than 1 L/kg (70 L).

Conversely, a drug was classified as non-dialyzable if any of the following criteria were met:

- The molecular weight exceeded 2000 Daltons.
- Protein binding exceeded 95%.
- The volume of distribution exceeded 3 L/kg (210 L).

In cases where the drug did not clearly fit into either the dialyzable or non-dialyzable category, it was categorized as ‘unable to determine’. Additionally, a drug was considered non-dialyzable if the label explicitly stated that the drug was not eliminated renally or was non-dialyzable. Furthermore, therapeutic proteins, such as insulin or monoclonal antibodies, were consistently categorized as non-dialyzable. Although some compounds which do not meet our definition of dialyzable may be cleared from the blood by some dialytic methods, we deliberately chose a conservative definition.

Once the data collection was complete, each individual conducted a quality check on their respective data for robustness. Finally, a single reviewer completed the final inspection and analysis of the data. This was undertaken with the specific aim of mitigating any discrepancies in the data, such as dialyzability, which inherently involved some level of judgement, to ensure consistency and integrity of the data collection process. Data analysis was conducted through the application of descriptive statistics.

## Results and Discussion

A total of 203 drugs were approved in the US during the selected years. Among these, 12 drugs had been discontinued or withdrawn from the market (and thus, the labels were not available through DailyMed), leaving 191 US labels available for review. Within this subset, 122 labels included an overdosage section. In parallel, it was observed that out of the 203 drugs approved in the US, 84 lacked a corresponding EPAR. Consequently, 119 EU labels were available for review.

### General Findings

In the years 2000–2001 and 2010–2011, a substantial majority of US approved drugs, specifically 91% (41/45) and 87% (40/46) respectively, had an overdosage section within their labeling, in contrast to 41% (41/100) of labels which were approved between 2020 and 2021. This finding may reflect the length of time the product has been on the market (i.e., newer products not yet having any overdoses reported), or other factors which were not investigated in our analysis, such as the specific characteristics of the drug product, and the method and setting of administration (e.g., administered by a HCP in hospitals or other healthcare settings). In contrast, the inclusion of an overdose section in the EU labels was 100% (119/119). This observation is expected given that overdose information is mandatory in the EU SmPC [3].

### Overdosage Signs and Symptoms

In the evaluated US and EU labels, experiences from clinical studies involving doses higher than those approved were described at a similar rate, 21% (26/122) and 29% (34/119) respectively. US labels more frequently listed adverse drug reactions (ADRs) from these studies compared to the EU labels. Specifically, 50% (13/26) of US labels contained ADRs from clinical studies that employed doses higher than the approved dose while only 21% (7/34) of EU labels included such information. The inclusion of information in the overdose section of labeling from clinical studies employing supratherapeutic doses is not addressed by either US or EU regulations or guidance documents. On one hand, presenting ADRs from these studies may provide useful information to prescribers on potential signs and/or symptoms of overdose. Also, if no ADRs are observed with doses several-fold higher than the therapeutic dose (i.e., the drug is known to have a high therapeutic index), this information can be of value to HCPs in the event of an overdose. On the other hand, if the overdosage section states that higher doses were studied, and no ADRs are presented and there is uncertainty regarding the safety profile at high doses, this could provide a false sense of assurance in the overdose setting or suggest safety of unapproved higher doses. Regulatory guidance to help navigate and establish a balanced approach in this matter may be of benefit.

A similar proportion of US (12%, 15/122) and EU labels (15%, 18/119) stated that the adverse reactions anticipated from a drug overdose are expected to be consistent with the general safety profile or the pharmacologic effects of the drug. These statements may help the prescriber in evaluating the potential medication overdose risks, as alignment between the anticipated ADRs and the established safety profile could facilitate an assessment in the event of an overdose.

More US labels (62%, 76/122) in contrast to EU labels (44%, 52/119) included information regarding specific ADRs in the overdosage section. The increased prevalence of reporting specific ADRs in US labels may be associated with the practice of excluding the overdosage section when relevant ADRs data are absent. Both the US and EU labels included information regarding single as well as multiple dose overdose experiences. Nonetheless, a significant majority of labels (approximately 75%) did not explicitly specify the number of doses in these instances.

Among the labels that included ADRs within the overdosage section, in a substantial majority of US and EU labels [95% (72/76) and 96% (50/52), respectively], these ADRs were included within other sections of the label. However, newer US labels also tended to incorporate ADRs exclusive to overdosage; that is, the ADRs were not documented in other sections of the label. In the years 2000–2001 and 2010–2011, 30% (9/30) and 46% (11/24) of US labels included ADRs exclusive to overdose, whereas this percentage increased to 73% (16/22) between 2020 and 2021. No equivalent observation was identified in the EU labels. Additional guidance may be beneficial in clarifying whether ADRs which are already listed in other sections of the label should be repeated in the overdosage section.

Information pertaining to risk factors associated with overdose was not commonly provided, with only 6% (7/122) of US labels and 3% (3/119) of EU labels including them. A description of these risk factor statements is described in Table 4. The US regulations do not make any reference for the inclusion of information on risk factors for overdose, and in the EU, this information is only mentioned in the SmPC training presentation [6]. Given the results, supplementary EU and US guidance on the inclusion of risk factor information associated with overdose may be of benefit. Understanding the risk factors for overdose allows HCPs to identify patients who may be at a higher risk of overdose based on various factors such as a history of substance abuse, mental health issues or certain medical conditions (e.g., renal impairment), enabling targeted interventions and closer monitoring for those at greater risk.

**Table 4:** Statements related to risk factors for overdoses

US (*n* = 7, 6%)	EU (*n* = 3, 3%)
Improper dose preparation	Co-administration with ketoconazole
Overdose with concomitant CNS depressants may be fatal; withdrawal seizures are more likely with use of flumazenil in patients with epilepsy	During re-initiation of treatment, monitor for bradycardia & AV block
High plasma drug levels or inadvertent subarachnoid injection	Renal impairment
Product is highly concentrated– be careful with dose calculation, especially for children	
Failure to adjust dose in patients with renal impairment has resulted in overdose	
Risks are related to increased radiation exposure and long-term risk of neoplasia	

Population-specific data were presented in a limited percentage of labels, accounting for 6% of both US (7/122) and EU (7/119) labels. In most instances, these data pertained to experiences or precautions specifically related to pediatric patients. The EU NtA provides clear guidance regarding the incorporation of pediatric considerations for this section of the label, including explicit instructions on their presentation i.e., “*should be a sub-section entitled pediatric population*”. This guideline also suggests including additional information pertaining to special populations, such as individuals with renal impairment. Despite existing guidelines recommending its inclusion, only 6% of EU labels incorporated population-specific data. In contrast, the US regulations do not mention including population-specific data in this section. Consequently, there may be benefit in providing supplementary guidance in this regard.

None of the US or EU labels incorporated in vitro data in the overdosage section of labeling. This observation aligns with the EU NtA guideline, wherein it is recommended that the description of the “*management of overdose in man”* be described, with in vitro data not explicitly recommended. Within the US regulations, the use of in vitro data is stated to be discretionary with the phrase “*may be*” employed where “*human data are unavailable*”. Consequently, the absence of in vitro data in the label is consistent with the regulatory discretion afforded by the US regulations.

Animal data were provided in 7% (9/122) of US labels, and 3% (3/119) of EU labels. The inclusion of animal data in only 3% of evaluated EU labels is consistent with the NtA guideline, wherein it is recommended that the description of the “*management of overdose in man*” be stated, with the inclusion of animal data not explicitly stated. In the SmPC Training Presentations FAQs, it states that *“due to limitations of interspecies extrapolation, non-clinical data is generally not mentioned in this section. Nevertheless, non-clinical data could provide support to observation made in the clinic”.* In five of the nine US labels that included animal data, corresponding human data or expected human responses were also included. Within the US regulations, the inclusion of animal data is stated to be discretionary with the phrase “*may be”* employed where “*human data are unavailable*”. Hence, this approach prompts questions regarding the rationale underpinning the inclusion of animal data within the labels where human data were available. The somewhat ambiguous language used in the US regulations, stating that animal data “*may be used*”, underscores the need for further clarification in a guidance document. Lethal dose-50 (LD50) data were identified in two US labels which were initially approved in the 2000–2001 time period. However, one of these labels was in the Physician’s Labeling Rule (PLR) format, and thus should follow the 21 CFR 201.57 requirements which does not include LD50. No LD50 data were documented within the EU labels.

### Overdosage Treatment

The EU labels (82%, 97/119) were more likely to present non-specific treatment recommendations within the overdose section, in contrast to the US labels (64%, 78/122). This difference may reflect the mandatory nature of the overdose section within EU labeling requirements. There is a need for additional guidance regarding the inclusion of non-specific treatment recommendations and their relevance to HCPs (e.g., statements such as ‘*Patients should be monitored for signs or symptoms of overdose and treatment provided as appropriate*’). To address this need, it is recommended that further insight be provided on when and how to present such information since the lack of specificity and tailored guidance in non-specific treatment recommendations may not be of clinical utility to HCPs in overdose situations.

39% (47/122) of US labels and 28% (33/119) of EU labels offered specific treatment and/or monitoring recommendations. In 25 instances, the drugs under consideration received approval in both the US and EU. The comparative analysis of recommendations for these drugs revealed the following:

- Recommendations were identical in both regions (*n* = 9).
- The EU label provided more comprehensive information (*n* = 9).
- The US label provided more comprehensive information (*n* = 2).
- Regional labels provided different information (*n* = 5).

No instances of contradictory information were identified throughout the examination. Regulatory guidance regarding the level of detail required for the treatment of overdoses may be of benefit. Specifically, when no antidote or reversal agent, such as naloxone or flumazenil, is available, specific treatment protocols may be difficult to describe and may go beyond what a single MAH can provide. In instances where the EU or US labels provided more extensive information, some of the additional information could be considered “medical practice”. Again, regulatory guidance in this area could be of benefit.

Drug dialyzability was addressed in only 37% (45/122) of US labels, despite regulatory requirements apparently mandating its inclusion. Drug dialyzability was addressed in 26% (31/119) of the EU labels. Drug dialyzability is only mentioned in the SmPC training presentations, which includes illustrative examples of the overdosage section. Notably, in two cases (1 from each of the US and EU labels), the drug met our definition of being ‘dialyzable’, but this was not mentioned in the overdose section. Understanding whether a drug is dialyzable is important for optimizing treatment strategies in overdose situations. It allows HCPs to consider dialysis as a targeted intervention to enhance drug elimination, potentially improving patient outcomes and reducing the risk of serious complications. In some cases (e.g., for therapeutic proteins), it may have been assumed that HCPs will know that the drug is not dialyzable. Additional guidance may be beneficial on the inclusion of information related to dialyzability of the drug, and the elimination half-life of a drug within the overdosage section. The elimination half-life helps in predicting how long it will take for the drug to be cleared from the body, or to lower to “non-toxic” concentrations.

Gastric lavage was recommended in 6% (7/122) of US and 3% (4/119) of EU labels. The presence of this recommendation in only 3% of EU labels is notably limited, aligning with the absence of a regulatory mandate for its inclusion. In contrast, the US regulations do refer to gastric lavage, albeit framed as an example of a specific measure to decontaminate the gastrointestinal tract to reduce further drug absorption, rather than as an explicit requirement for inclusion. Given the uncertainty regarding the effectiveness of gastric lavage as a gastrointestinal decontamination method [11], regulatory guidance on when to refer to this treatment may be helpful.

The analysis revealed that 20% (25/122) of the US labels referenced Poison Centers in contrast to 3% (4/119) of the EU labels. The low prevalence of such references within EU labels aligns with expectation, given the absence of regulations or guidance stipulations pertaining to the incorporation of these centers in the label. The inclusion of Poison Center references in only 20% of US labels is of interest. While Poison Centers are indeed mentioned in US regulations, their incorporation is currently presented as an illustrative example, rather than being specifically mandated. From an EU perspective, neither the regulation nor available guidances explicitly mention the need to include a reference to Poison Centers in labeling. However, the identification of EU labels that do incorporate such references highlights a potential discrepancy in practice. To ensure consistency, it is recommended that supplementary guidance be provided. Such guidance could address not only when to include this information but also its relevance and utility to prescribers. It may be that HCPs routinely call Poison Centers to obtain more information than what is provided in the label.

### Limitations of Our Research

There are several limitations to this research. We used a definition for “*dialyzable drugs”*, but we did not verify whether the identified drugs are indeed dialyzable, highlighting a potential discrepancy between the defined category and the actual dialyzability of the drugs included in the analysis. Additionally, the research did not incorporate HCPs feedback, representing a limitation in obtaining valuable insights for potential enhancements to overdose-related information. Furthermore, the research did not analyze the inclusion of information related to concentration-dependent toxicity. Finally, the research did not extend to the evaluation of the initial labels proposed by MAHs to the FDA or EMA. The focus solely relied on publicly available documents, and this may limit our understanding of the full spectrum of information provided by MAHs and the Regulatory Authorities during the regulatory submission and approval process. These limitations highlight areas where the research could be expanded or refined in the future.

## Conclusions

The research findings reveal that although the content of the overdosage section within the US and EU labels generally aligns with regional expectations and regulatory requirements, there are areas which may benefit from additional regulatory guidance. Addressing these areas may assist in making information relevant to overdoses more easily accessible to clinicians (e.g., how to succinctly provide pharmacokinetic data, or adverse reaction data in the overdose section of the label without fully duplicating information from other sections of the label, or methods to frame data from specific populations such as children). Regulatory guidance to MAHs on how to develop this section of labeling (e.g., when / how to include information from studies which used supratherapeutic doses, or presentation of non-specific treatment recommendations) would help to ensure a consistency of approach, which may also be of benefit to clinicians.

## Data Availability

Data is provided within the manuscript.
